# Rational Design of a Functional Fatty Acid Component for Alleviating Western Diet-Induced Insulin Resistance and Glycolipid Metabolism Disorders

**DOI:** 10.3390/foods15061016

**Published:** 2026-03-13

**Authors:** Qingyun Guan, Xia Pi, Feixue Wu, Chunmei Li

**Affiliations:** 1 College of Food Science and Technology, Huazhong Agricultural University, Wuhan 430070, China; 2 Key Laboratory of Environment Correlative Food Science, Ministry of Education, Huazhong Agricultural University, Wuhan 430070, China

**Keywords:** fatty acids, insulin resistance, glycolipid metabolism, functional fatty acid component, gut microbiota, *Akkermansia*

## Abstract

This research aimed to systematically investigate the regulatory effects of six key fatty acids and rationally designed a functional fatty acid component (FFAC) to alleviate palmitic acid (PA)-induced glycolipid metabolism disorders and insulin resistance (IR) in HepG2 cells and Western diet-induced IR in the C57BL/6 mice model. In vitro experiments showed that saturated fatty acids such as PA and stearic acid (SA) induced concentration-dependent cytotoxicity and IR in HepG2 cells, while unsaturated fatty acids, including palmitoleic acid (POA), oleic acid (OA), linoleic acid (LA), and α-linolenic acid (ALA), enhanced cell viability and exerted protective effects. Based on the principle of balanced fatty acid ratio and the obtained cell experimental results, FFAC was designed as PA:SA:POA:OA:LA:ALA = 4:1:1:4:4:1 and formulated using dietary oils. In vivo, a 13-week dietary intervention revealed that FFAC substitution mitigated Western diet-induced weight gain, systemic IR, serum lipid disorders, and hepatic steatosis in mice. Mechanistically, FFAC restored the IRS1/PI3K/Akt/GSK3β insulin signaling pathway in HepG2 cells and reshaped gut microbiota by enriching beneficial genera such as *Akkermansia*. These findings demonstrated that FFAC effectively alleviates diet-induced metabolic disorders through multiple pathways, highlighting the potential of rationally designed dietary fatty acid compositions in managing metabolic disorders.

## 1. Introduction

Insulin resistance (IR) has emerged as a critical focus in food and nutritional research due to the escalating global burden of metabolic diseases [1,2]. Defined as a diminished biological response to normal circulating insulin concentrations, IR represents a fundamental defect in the maintenance of glucose and lipid homeostasis. Consequently, it is a key predictor for the development of Type 2 Diabetes Mellitus, a chronic condition characterized by hyperglycemia resulting from both insulin resistance and impaired insulin secretion [3,4]. Given this pathogenic role, strategies aimed at managing IR are considered to be approaches for improving metabolic health in at-risk individuals [5,6].

The pathogenesis of IR is tightly associated with fatty acid metabolism, influenced by systemic concentrations and especially dietary intake [5,6,7,8]. Obesity, a primary risk factor for IR, is associated with elevated plasma free fatty acids (FFAs). These excess FFAs act as active pathogenic drivers, contributing to ectopic lipid accumulation, provoking inflammatory cascades, and inducing mitochondrial as well as endoplasmic reticulum stress within insulin-sensitive tissues [7,9,10]. Critically, the metabolic effects of fatty acids are determined by their chemical structures [11]. Dietary fatty acid composition directly shapes plasma and cellular lipid profiles [12]. Substantial evidence implicates saturated fatty acids (SFAs), notably palmitic acid (PA, C16:0), as potent inducers of IR through fostering the accumulation of diacylglycerols (DAG) and ceramides, activating pro-inflammatory signaling (e.g., NF-κB), and disrupting insulin signal transduction [13,14,15]. In contrast, monounsaturated fatty acids such as oleic acid (OA, C18:1 n-9) often attenuate PA-induced inflammation, promote lipid oxidation and safe storage as triglycerides, and thereby enhance insulin sensitivity [7,16,17]. The roles of polyunsaturated fatty acids are complex, but those of long-chain ω-3 types such as eicosapentaenoic acid and docosahexaenoic acid are recognized for their anti-inflammatory and insulin-sensitizing properties, mediated through receptors such as GPR120 and peroxisome proliferator-activated receptors [9,18,19].

Although the individual effects of PA and OA are well-documented currently [13,14,15,16,17,20], a systematic comparative evaluation involving the effect of stearic acid (SA, C18:0), palmitoleic acid (POA, C16:1 n-7), linoleic acid (LA, C18:2 n-6), and α-linolenic acid (ALA, C18:3 n-3) on hepatic insulin sensitivity has not been fully elucidated. Especially, the potential synergistic effects and mechanistic investigations of these fatty acids were less explored [15]. Moreover, to the best of our knowledge, no study to date has rationally designed a “functional fatty acid component (FFAC)” based on the evaluated synergistic interactions and validated it in both in vitro and in vivo models, while a significant gap remains in the integrating mechanistic cell studies with a translational dietary oil formulation, offering a composition-driven strategy for metabolic disorders.

To address these gaps, the present study aims to rationally design a functional fatty acid component for the prevention and management of glycolipid metabolism disorders and IR. First, a comparative analysis of six key dietary fatty acids (PA, SA, POA, OA, LA, and ALA) on lipid metabolism and insulin signaling in human HepG2 cells was performed. Informed by these results, a specific blend of these fatty acids was then formulated to optimally counteract PA-induced insulin resistance in vitro and elucidate its underlying mechanisms. Finally, this optimized component was translated into a dietary oil formulation and evaluated for its efficacy in enhancing whole-body insulin sensitivity in mice fed a high-fat/high-sugar Western diet. This research bridges a critical translational gap between mechanistic nutritional biochemistry and practical dietary intervention, thereby proposing a novel, composition-driven strategy for mitigating IR.

## 2. Materials and Methods

### 2.1. Materials and Reagents

Dulbecco’s Modified Eagle Medium (DMEM) and fetal bovine serum (FBS) were obtained from GIBCO BRL (Grand Island, NY, USA). Penicillin/streptomycin was purchased from Procell Co., Ltd. (Wuhan, China). The phosphate-buffered saline (PBS) and fatty acid-free bovine serum albumin (BSA) were bought from Solarbio Science and Technology Co., Ltd. (Beijing, China). 3-(4,5-dimethyl-2-thiazolyl)-2,5-diphenyl tetrazolium bromide (MTT) and Bicinchoninic Acid (BCA) protein assay kit were provided by Beyotime Biotechnology Co., Ltd. (Shanghai, China). Insulin was obtained from Novo Nordisk Pharmaceutical Co., Ltd. (Tianjin, China). All the primary antibodies were obtained from Cell Signaling Technology (Danvers, MA, USA), while the secondary antibody was obtained from ABclonal Biotechnology Co., Ltd. (Wuhan, China). The glucose oxidase kit and the assay kits for serum/hepatic triglycerides (TG), total cholesterol (TC), FFAs, high-density lipoprotein-cholesterol (HDL-C), low-density lipoprotein-cholesterol (LDL-C), alanine aminotransferase (ALT), and aspartate aminotransferase (AST) were purchased from Nanjing Jiancheng Bioengineering INST. (Nanjing, China). The human liver glycogen enzyme-linked immunosorbent assay (ELISA) kit was obtained from Enzyme-linked Biotechnology Co., Ltd. (Shanghai, China). The DNA extraction kit was obtained from Omega Bio-tek, Inc. (Norcross, GA, USA), AxyPrep DNA gel extraction kit was purchased from Axygen Biosciences (Union City, CA, USA). RIPA lysis buffer, SDS-PAGE gel kit, protease inhibitor, and 4% paraformaldehyde were provided by Sevier Biotechnology Co., Ltd. (Wuhan, China). All diets were obtained from Ready Dietech (Shenzhen, China). Other chemical reagents used were of analytical or cell culture grade from Sinopharm Chemical Reagent Co., Ltd. (Shanghai, China). Ultrapure water was generated using the Milli-Q Plus system (Billerica, MA, USA).

### 2.2. Cell Culture and Animal Models

The human hepatocellular carcinoma cell line HepG2 cells were selected due to their well-differentiated hepatocyte-like phenotype and extensive use in studies of lipid metabolism and insulin signaling [21,22,23], and purchased from Shenghang Biological Technology Co., Ltd. (Nanjing, China). Cells were maintained in the DMEM supplemented with 10% FBS and 1% penicillin/streptomycin at 37 °C in a humidified incubator with 5% CO_2_. All cell culture experiments were performed in triplicate, independent experiments. The data are presented as the mean ± SD (n = 3). Otherwise, the fatty acids were complexed with fatty acid-free BSA for delivery in cell culture experiments, based on the preliminary experiments, which showed that BSA at concentrations below 120 μM had no significant effect on cell viability (*p* > 0.05), consistent with a previous study [24]. The BSA concentration used in the cell experiments was set as 20% of that of fatty acids, which was lower than 120 μM.

All animal procedures were approved by the Experimental Animal Care and Use Committee of Huazhong Agricultural University (Ethics Number: 202502270008). Male C57BL/6 mice (6 weeks old, 23.4 ± 0.7 g) were chosen because they are a standard model for diet-induced obesity and insulin resistance, and are susceptible to high-fat/high-sugar diets [15,17,25], and obtained from the Experimental Animal Center of Huazhong Agricultural University (Wuhan, China) and housed under specific pathogen-free conditions with a 12 h light–dark cycle, temperature maintained at 24–26 °C, and humidity at 50–60%. After adapted for one week, all the mice were randomly assigned to 3 groups (n = 10 per group) as follows: Group C (Control) with a standard chow diet; Group W (Western diet) with a high-sugar and high-fat diet (the fatty acid composition was 95.2% anhydrous butteroil and 4.8% soybean oil, as reported previously [26]); Group WB with a high-sugar and high-fat diet substituted with the optimized balanced functional fatty acid component (PA:SA:POA:OA:LA:ALA = 4:1:1:4:4:1) formulated using dietary oils (i.e., 29.0% sunflower oil, 25.5% anhydrous butteroil, 24.0% sea buckthorn fruit oil, 10.0% lard, and 11.5% flaxseed oil). The total energy density of these diets was controlled at 4.7 kcal/g. The animal experiment lasted 13 weeks, based on the pre-experiments and literature reports [27,28,29]. All animal procedures were conducted in accordance with institutional guidelines. No animals were excluded from the analysis; all data points were included in the final statistical analysis (n = 10).

### 2.3. Cell Viability Assay

Cell viability was measured using the MTT assay. HepG2 cells (5 × 10^4^ cells/well) were seeded in 96-well plates. At approximately 80% confluence, cells were treated with fatty acids at concentrations ranging from 0 to 600 μM for 24 h, based on the pre-experiments and literature reports [21,30]. Subsequently, the medium was replaced with 150 μL MTT solution (0.5 mg/mL) and incubated for 4 h. The formazan crystals formed were dissolved in DMSO, and the absorbance was measured at 490 nm. Viability was expressed as a percentage relative to the untreated control cells.

### 2.4. Lipid Accumulation and Triglyceride Content Quantification

Lipid accumulation was visualized by Oil Red O staining. HepG2 cells (6 × 10^5^ cells/well in 12-well plates) were treated with fatty acids for 24 h, fixed with 4% paraformaldehyde for 30 min, and stained with Oil Red O solution for 30 min. The stained lipid droplets were dissolved in 1 mL of isopropanol on ice for 30 min, and the absorbance of the eluate was measured at 492 nm.

Intracellular TG content was quantified biochemically. After treatment, cellular lipids were extracted from HepG2 cells using 1 mL/well of n-hexane/isopropanol (3:2, *v*/*v*) for 1 h. The extracts were dried under nitrogen gas, reconstituted in 50 μL of isopropanol, and the TG content was measured using a commercial assay kit. Results were normalized to total cellular protein concentration determined by a BCA assay and expressed as mmol/g protein.

### 2.5. Glucose Consumption and Glycogen Content Measurement

The glucose consumption of HepG2 cells (3 × 10^5^ cells/well in 24-well plates) treated with fatty acids for 24 h was determined by measuring the glucose concentration in the medium before and after insulin stimulation with 150 nM insulin for 3 h, using a commercial glucose assay kit. Values were corrected for cell number based on parallel MTT assays.

Glycogen content was measured from lysates prepared via freeze–thaw cycles of HepG2 cells (1.2 × 10^6^ cells/well in 6-well plates) treated with fatty acids for 24 h, using a commercial ELISA kit, and normalized to total protein and expressed as mg/mg protein.

### 2.6. Western Blotting

HepG2 cells (1.2 × 10^6^ cells/well in 6-well plates) were divided into three treatment groups for 24 h: control, model (treated with 100 μM palmitic acid), and functional fatty acid component (FFAC; treated with the optimized functional fatty acid component while the concentration of PA was 100 μM). Following treatment, cells were stimulated with 150 nM insulin for 20 min and lysed on ice for 30 min using 300 μL of lysis buffer/well (RIPA supplemented with protease and phosphatase inhibitors). The proteins were separated by SDS-PAGE using 8% separating and 5% stacking gels, transferred to PVDF membranes, and probed with specific primary antibodies overnight at 4 °C. After incubation with HRP-conjugated secondary antibodies for 50 min, protein bands were visualized using an enhanced chemiluminescence reagent, imaged with an Odyssey Fc Imaging System (LI-COR Biosciences, Lincoln, NE, USA), and quantified using ImageJ software (version 1.54f).

### 2.7. Analysis of Fatty Acid Composition in Dietary Oils

The fatty acid profiles of the dietary oils used in animal feeds were determined using an Agilent 6890N gas chromatograph (Agilent Technologies, Santa Clara, CA, USA). Briefly, 50 mg of oils were subjected to 2 mL of 0.4 M KOH-CH_3_OH and 2 mL of methanol. Fatty acid methyl esters were extracted with 2 mL of n-hexane. Analysis was performed using an HP-88 capillary chromatographic column (30 mm × 0.25 mm, 0.2 μm) after a 0.22 μm filter membrane. Methyl ester of octadecanoic acid was used as an internal standard. Specific conditions were as follows: injection volume: 1.0 μL; injector/detector temperature: 250 °C; split ratio: 50:1; carrier gas: nitrogen at 1.0 mL/min. The temperature program was as follows: initial hold at 100 °C for 0.5 min, increase to 194 °C at 30 °C/min (hold 3.5 min), and then increase to 224 °C at 5 °C/min (hold 1 min).

### 2.8. Animal Experiment

For the oral glucose tolerance test, mice fasted for 12 h received glucose (2 g/kg body weight) by oral gavage. Blood glucose was measured from the tail vein blood at 0, 15, 30, 60, 90, and 120 min using a glucometer (GA-3, Sinocare, Changsha, China). For the insulin tolerance test, mice fasted for 6 h received an intraperitoneal injection of insulin (0.75 U/kg body weight), and blood glucose was monitored at the same time points.

At the end of the 13-week feeding period, the blood was collected after the mice were decapitated, and serum was prepared for analysis. Subcutaneous inguinal white adipose tissue (iWAT), epididymal white adipose tissue (eWAT), and perirenal fat were carefully dissected and weighed immediately. Serum levels of TG, TC, HDL-C, LDL-C, FFAs, ALT, AST, and insulin were measured using assay kits. For hepatic lipid analysis, 50 mg of liver was homogenized in 450 μL of PBS on ice, followed by centrifugation at 2500× *g* for 10 min at 4 °C, and the supernatant was assayed for TG, TC, HDL-C, and LDL-C content. Moreover, the colon content samples were collected under sterile conditions and stored at −80 °C. Total microbial DNA was extracted using a corresponding kit, assessed by 1% agarose gel, and the hypervariable V3-V4 region of the 16S rRNA gene was amplified via PCR on a GeneAmp 9700 System (ABI, Los Angeles, CA, USA). Amplicons were separated by 2% agarose gel electrophoresis, purified using an AxyPrep DNA gel extraction kit, and quantified with a QuantiFluor-ST blue fluorescence system (Promega Co., Madison, WI, USA). Equimolar pooled amplicons were subjected to paired-end sequencing on the Illumina MiSeq platform (San Diego, CA, USA). The resulting reads were analyzed at Majorbio Bio-Pharm Technology Co., Ltd. (Shanghai, China).

### 2.9. Statistical Analysis

The obtained data were expressed as mean ± standard deviation. Statistical comparisons among multiple groups were performed using one-way analysis of variance (ANOVA) followed by Waller-Duncan’s post hoc test. The *p* < 0.05 was considered statistically significant. Analyses were conducted using SPSS 20.

## 3. Results and Discussion

### 3.1. Cytotoxic Effects of SFAs and Protective/Proliferative Effects of UFAs on Cell Viability

**Figure 1:**
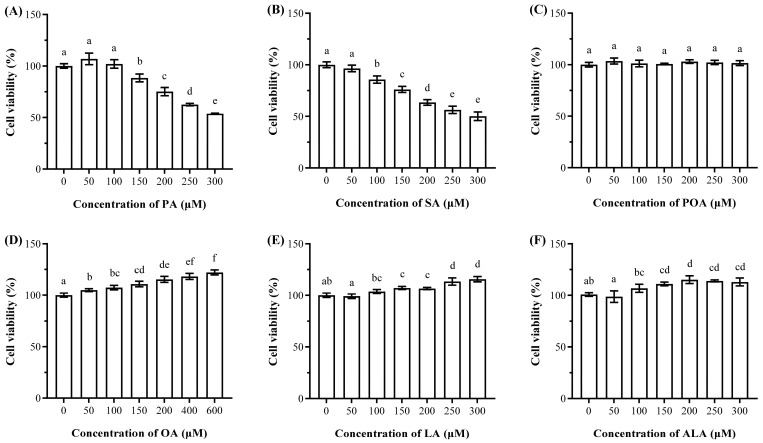
Viability of HepG2 cells treated 24 h by fatty acids, including palmitic acid (PA, (**A**)), stearic acid (SA, (**B**)), palmitoleic acid (POA, (**C**)), oleic acid (OA, (**D**)), linoleic acid (LA, (**E**)) and α-linolenic acid (ALA, (**F**)) at various concentrations (0–600 μM) (n = 3). Bars with different letters are significantly different from each other (*p <* 0.05).

To determine the appropriate concentration range for subsequent experiments and establish a baseline for cellular health under fatty acid exposure, MTT assays were performed following 24 h exposure to fatty acids, as illustrated in Figure 1. Overall, SFAs, including PA and SA, demonstrated obvious cytotoxicity at higher concentrations, while the tested UFAs did not impair cell viability within the examined concentration ranges (0–600 μM). Particularly, PA (or SA) significantly reduced viability at concentrations ≥ 150 µM (or ≥100 µM), and the viability of PA-treated and SA-treated cells dropped to approximately 50% at 300 µM (*p* < 0.05). In contrast, POA had no significant effect on viability up to 300 µM (*p* > 0.05). Notably, OA significantly increased viability at 50 µM, while LA and ALA significantly upregulated viability at 150 µM (*p* < 0.05). These results indicated that excessive SFAs induced concentration-dependent cytotoxicity, whereas UFAs could offer a protective or proliferative effect under these conditions, consistent with previous studies [13,14,31].

### 3.2. Effects of Fatty Acids on HepG2 Cell Glycolipid Metabolism

#### 3.2.1. SFAs and UFAs Promote Lipid Accumulation Through Divergent Metabolic Fates

**Figure 2:**
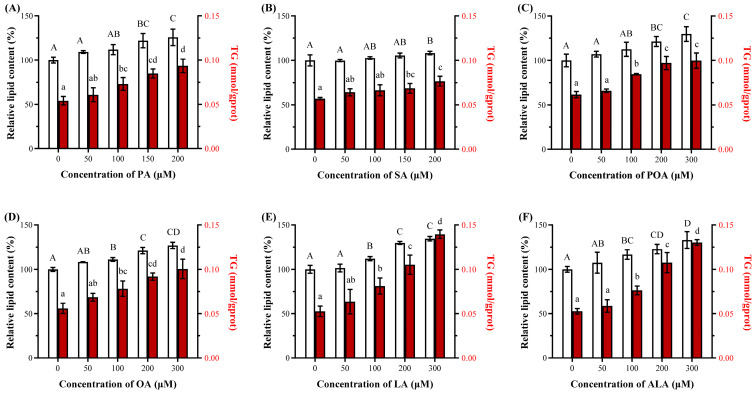
The relative lipid and triglyceride (TG) content of HepG2 cells treated over 24 h by fatty acids, including palmitic acid (PA, (**A**)), stearic acid (SA, (**B**)), palmitoleic acid (POA, (**C**)), oleic acid (OA, (**D**)), linoleic acid (LA, (**E**)) and α-linolenic acid (ALA, (**F**)) at various concentrations (0–300 μM) (n = 3). Bars with different letters are significantly different from each other (*p <* 0.05).

Whether these fatty acids influenced HepG2 cell lipid metabolism was further investigated by Oil Red O staining and TG quantification. As demonstrated in Figure 2, treatment with all six fatty acids significantly increased intracellular lipid accumulation beyond specific concentrations (i.e., PA ≥ 150 µM; SA ≥ 200 µM; POA ≥ 200 µM; OA, LA, ALA ≥ 100 µM; *p* < 0.05). Quantitative analysis showed that the lipid content relatively increased 25.8% (PA), 8.3% (SA), 21.4% (POA), 21.2% (OA), 29.9% (LA), and 22.9% (ALA) at 200 µM relative to the control group. Correspondingly, SA induced the weakest TG content increase, whereas UFAs like LA triggered the most substantial TG accumulation (16.5% vs. 54.5% increase at 100 µM). Critically, UFAs triggered the most substantial TG deposition without reducing cell viability, indicating that lipid accumulation itself is not inherently cytotoxic, while the differential metabolic partitioning of fatty acids underlies the disparity between lipid accumulation and cell survival outcomes [14,16]. Aligning with previous studies, incorporation of fatty acids into relatively inert TG pools could be a protective detoxification mechanism, whereas SFAs (e.g., PA) might favor the accumulation of harmful intermediates such as DAG and ceramides when their oxidative capacity is exceeded [13,14], linking their metabolic fate to the cytotoxic profile.

#### 3.2.2. SFAs Potently Induce Insulin Resistance

**Figure 3:**
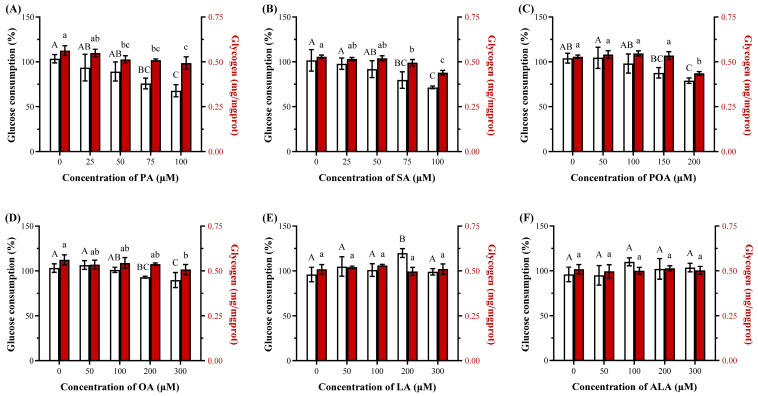
The relative glucose consumption and glycogen content of HepG2 cells treated for 24 h by fatty acids, including palmitic acid (PA, (**A**)), stearic acid (SA, (**B**)), palmitoleic acid (POA, (**C**)), oleic acid (OA, (**D**)), linoleic acid (LA, (**E**)) and α-linolenic acid (ALA, (**F**)) at various concentrations (0–300 μM), followed by an insulin stimulation at 100 nM for 3 h (n = 3). Bars with different letters (lowercase/uppercase) are significantly different from each other (*p <* 0.05).

Given the central role of hepatic insulin sensitivity in metabolic health [32], whether the observed differences extended to this functional point was evaluated via glucose consumption and glycogen content under insulin stimulation. As shown in Figure 3, SFAs (i.e., PA and SA) were potent inducers of insulin resistance in HepG2 cells, evidenced by the significantly reduced glucose consumption at concentrations as low as 75 µM (26.7% and 21.7%, respectively) and significantly decreased cellular glycogen content at 50–75 µM (*p <* 0.05), which preceded their cytotoxic effects. In contrast, UFAs (e.g., POA and OA) induced resistance only at higher concentrations (over 150–200 µM), while LA and ALA did not impair sensitivity within the tested range (*p* > 0.05). The obtained data suggested that the propensity to disrupt insulin signaling is not directly linked to the magnitude of TG accumulation but might relate to the generation of specific lipid mediators [20,30,33]. Based on the significant reduction in insulin sensitivity without severe loss of viability, and combined with the data reported in the references [20,34], 100 µM PA was selected for establishing the insulin resistance HepG2 cell model in subsequent experiments.

### 3.3. Interactions Between Palmitic Acid and Other Fatty Acids in Modulating Cell Metabolism

#### 3.3.1. UFAs Exacerbate PA-Induced Lipid Accumulation

**Figure 4:**
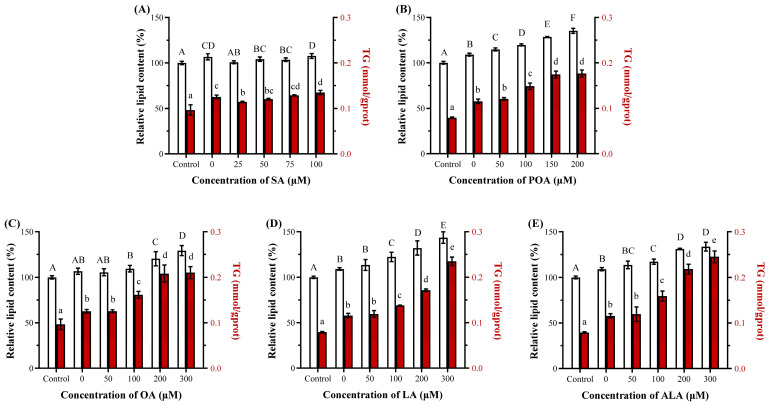
The relative lipid and triglyceride (TG) content of HepG2 cells treated 24 h by palmitic acid (PA, 100 μM) and fatty acids, including stearic acid (SA, (**A**)), palmitoleic acid (POA, (**B**)), oleic acid (OA, (**C**)), linoleic acid (LA, (**D**)) and α-linolenic acid (ALA, (**E**)) at various concentrations (0–300 μM) (n = 3). Bars with different letters (lowercase/uppercase) are significantly different from each other (*p* < 0.05).

To explore potential synergistic or antagonistic interactions of fatty acids, whether other fatty acids could modulate the metabolic dysfunction induced by PA was further investigated. As shown in Figure 4, co-treatment of PA with SA or various UFAs yielded contrasting results. Overall, UFAs (i.e., POA, OA, LA, and ALA) significantly exacerbated lipid (9.8%, 0.3%, 12.2%, and 7.6% at 100 μM, respectively) and TG (28.6%, 29.0%, 19.7%, and 37.6% at 100 μM, respectively) accumulation compared to PA alone (*p* < 0.05, Figure 4B–E). Conversely, co-treatment with PA and SA only demonstrated a minor increase in lipid load and TG content (0.9% and 7.8%, respectively), implying that excessive SFAs lead to the accumulation of harmful lipid metabolic products such as DAG in HepG2 cells instead of harmless TG synthesis [21,35]. This dissociation underscored a critical distinction: exacerbation of lipid accumulation by UFAs does not worsen, but instead ameliorates, PA-induced insulin resistance. Theoretically, UFAs exacerbate TG accumulation by serving as substrates for triglyceride synthesis via DGAT enzymes, thereby diverting PA from pathways leading to DAG and ceramide synthesis, which were considered the harmful lipid intermediates that disrupt insulin signaling, while TG storage is considered a protective buffering mechanism [13,15]. Further explained why the introduced UFAs worsen lipid load yet improve insulin sensitivity. Furthermore, UFAs may activate counter-regulatory pathways, such as POA, which could improve insulin sensitivity in muscle cells, while OA and EPA could mitigate oxidative and endoplasmic reticulum stress [36,37]. The protective TG accumulation observed with UFA co-treatment plays a direct role in this mitigation.

#### 3.3.2. UFAs Restores Glucose Metabolism in PA-Induced Insulin Resistance

**Figure 5:**
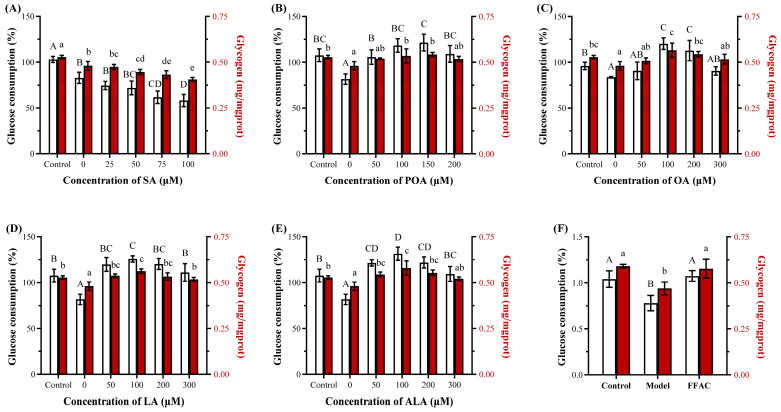
The relative glucose consumption and glycogen content of HepG2 cells treated 24 h by palmitic acid (PA, 100 μM) and fatty acids, including stearic acid (SA, (**A**)), palmitoleic acid (POA, (**B**)), oleic acid (OA, (**C**)), linoleic acid (LA, (**D**)), α-linolenic acid (ALA, (**E**)) at various concentrations (0–300 μM) and the designed functional fatty acid component (**F**), followed by an insulin stimulation at 100 nM for 3 h (n = 3). Bars with different letters (lowercase/uppercase) are significantly different from each other (*p <* 0.05).

Furthermore, whether UFAs could alter metabolic partitioning correlated with improvements in insulin signaling, as assessed by glucose metabolism, was investigated. The measured cellular glucose consumption and glycogen content after the co-treatment of fatty acids with PA are demonstrated in Figure 5. The co-incubation of SFAs (PA and SA) further reduced the insulin sensitivity, while UFAs at certain concentrations could improve PA-induced insulin resistance in HepG2 cells. In detail, the glucose consumption and glycogen content gradually decreased with the increase in SA concentration (reaching 30.0% and 15.4% at 100 μM), compared to the model group (*p* < 0.05). The cells co-treated by PA and UFAs (including POA, OA, LA, and ALA) at the concentration range of 0–100 μM demonstrated a significant increase trend in the glucose consumption and glycogen content, compared with the model group (*p* < 0.05). Especially, the measured glucose consumption and glycogen content values even reached or were higher than the level of the control group, although the concentration of UFAs was as low as 50 μM (*p* < 0.05), indicating that the introduced UFAs concurrently ameliorated PA-induced insulin resistance, restoring or even enhancing glucose consumption and glycogen synthesis. While the excessive fatty acids lead to an accumulation of lipids and hinder the conduction of the insulin signaling pathway, such as the ROS/c-Jun signaling pathway [36,37,38]. It has been reported that the OA can down-regulate intracellular ROS levels by reducing endoplasmic reticulum stress, while EPA reduces ROS production by inhibiting mitochondrial dysfunction induced by PA, consistent with the measured results [38]. Several studies exhibited that PA treatment damaged the insulin signaling pathway conduction and insulin-stimulated glycogen synthesis in C2C12 myotubes, and increased the phosphorylation of p38 mitogen-activated protein kinase and c-Jun N-terminal kinase in the myotubes, while POA improved insulin sensitivity of myotubes through activating macrophages and counteracting insulin resistance mediated by PA [10,36,39,40]. Overall, the introduced UFAs, even at low concentrations (e.g., 50 μM), might restore insulin-stimulated glucose uptake and glycogen synthesis by facilitating the partitioning of PA into neutral lipids (e.g., TG), and reducing ER stress and ROS production, thereby preserving insulin signaling integrity [20,41,42,43,44].

### 3.4. Rational Design of a Balanced Functional Fatty Acid Component

Synthesizing the above data and the dietary principle of balanced fatty acids intake (i.e., SFA:MUFA:PUFA ratio = 1:1:1) with attention to omega-6: omega-3 balance (4:1), an optimal ratio was designed [45,46,47]. Firstly, since SA, as a common SFA should not be overlooked, and the data showing that SA at a low dosage (25 µM) did not worsen PA-induced IR (concentration of PA: 100 µM), while higher dosages exacerbated it (Figure 5A); thus, a minimal SA level (PA:SA = 4:1) was retained to reflect realistic dietary proportions. As 100 µM OA co-treatment showed pronounced benefits, a PA:OA ratio of 1:1 was set (Figure 5C). Therefore, several equations have been addressed: PA + SA = 33.3%; POA + OA = 33.3%; LA + ALA = 33.3%; PA:SA = 4:1; PA:OA = 1:1; and LA:ALA = 4:1, the obtained a optimal fatty acid ratio as PA:SA:POA:OA:LA:ALA = 4:1:1:4:4:1, leading a proportions as 26.7%, 6.7%, 6.7%, 26.7%, 26.7% and 6.7% respectively. Subsequently, the effect of the designed FFAC on the glucose consumption and glycogen content in HepG2 cells was investigated under the same PA concentration (100 μM). After 24 h of treatment, the measured glucose metabolism (Figure 5F) showed that compared with the model group, the glucose consumption and glycogen content of the FFAC group increased by 38.0% and 22.9%, respectively (*p* < 0.05), approaching the level of the control group, indicating that the designed FFAC effectively avoided the PA-induced insulin resistance in HepG2 cells.

### 3.5. FFAC Ameliorates Insulin Resistance by Restoring the IRS1/PI3K/Akt/GSK3β Signaling Pathway

**Figure 6:**
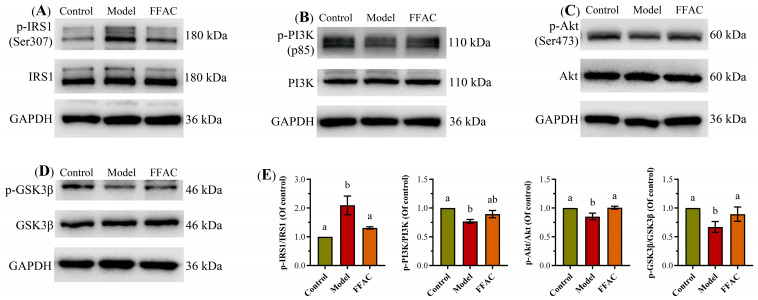
The Western blot images of p-IRS1/IRS1 (**A**), p-PI3K/PI3K (**B**), p-Akt/Akt (**C**), and p-GSK3β/GSK3β (**D**) levels and their relative levels (**E**) in HepG2 cells treated with control, model, and functional fatty acid component (n = 3). Bars with different letters are significantly different from each other (*p <* 0.05).

The molecular mechanisms underlying PA-induced insulin resistance in HepG2 cells and the corrective effects of FFAC were further investigated. Consistent with previous reports [10,39,48], treatment with PA (100 μM) significantly impaired the insulin signaling cascade through elevating the inhibitory phosphorylation of IRS1 (Ser307) by 209.3% compared to the control (as shown in Figure 6), a modification known to uncouple IRS1 from the insulin receptor and block downstream signaling. Consequently, the activation of pivotal downstream kinases was suppressed, as shown by reduced phosphorylation of the PI3K (p85) and Akt (Ser473). Since Akt serves as a central hub regulating multiple metabolic effects of insulin, its impaired activation culminated in a marked reduction in inhibitory phosphorylation on its substrate, glycogen synthase kinase 3β (GSK3β), to 66.9% of control levels. As phosphorylated (inactive) GSK3β normally permits glycogen synthase activation, this decrease directly correlates with the observed hindrance of glycogen synthesis, a key insulin-dependent hepatic function [25,49].

Mechanistically, our designed FFAC effectively counteracted the PA-induced dysfunction. Which significantly normalized the insulin signaling pathway, reducing the p-IRS1/IRS1 ratio to 130.5% and restoring the p-PI3K/PI3K and p-Akt/Akt ratios to 89.4% and 100.5% of control levels, respectively. The restoration of Akt activity was further confirmed by the recovery of GSK3β phosphorylation, thereby promoting glycogen synthase activity. These findings align with and extend previous reports indicating that prolonged PA exposure inhibits AKT phosphorylation and impairs insulin-stimulated glucose uptake [49], and that PA reduces levels of p-IRβ, p-IRS1 (Tyr612), p-Akt (Ser473), and p-GSK3β [10,25]. The restoration of the IRS1/PI3K/Akt/GSK3β pathway activity confirmed that the FFAC improved insulin sensitivity by enhancing proximal insulin signal transduction. In summary, the results delineated a clear hierarchy that SFAs like PA potently impaired insulin signaling, while the rationally designed FFAC could effectively counteract this dysfunction, thus underscoring the paramount importance of overall fatty acid composition, rather than merely total fat exposure, in maintaining metabolic homeostasis and insulin resistance.

### 3.6. Translational Formulation: Blending Dietary Oils to Achieve the Designed FFAC

To reach the optimal fatty acid ratio (i.e., PA:SA:POA:OA:LA:ALA = 4:1:1:4:4:1) as designed above, the main fatty acid components of six dietary oils, including anhydrous butteroil, lard, sea buckthorn fruit oil, sunflower oil, flaxseed oil, and soybean oil, were comprehensively detected as summarized in Table 1. The obtained results demonstrated that anhydrous butteroil was mainly composed of high levels of myristic acid (C14:0, 13.7%) and PA (C16:0, 40.9%), along with moderate amounts of OA (C18:1, 26.1%) and SA (C18:0, 12.9%). Lard exhibited considerable proportions of OA (37.5%), PA (30.5%), and SA (15.7%). Sea buckthorn fruit oil displayed a distinctive composition, with the highest content of POA (C16:1, 23.91%) among the oils tested, accompanied by substantial OA (32.5%) and PA (28.0%). Sunflower oil was predominantly rich in LA (C18:2, 67.6%), while flaxseed oil contained a remarkably high level of ALA (C18:3, 54.6%). Soybean oil, though analyzed for reference, presented a more balanced but less concentrated profile across multiple fatty acids and was not selected as a primary component due to its lower specificity for the target fatty acids. Based on the quantified fatty acid compositions, the blending calculation demonstrated that the desired profile could be attained by mixing 25.5% anhydrous butteroil, 10.0% lard, 24.0% sea buckthorn fruit oil, 29.0% sunflower oil, and 11.5% flaxseed oil, which was named as “functional fatty acid component (FFAC) for animal experiments”. FFAC effectively leverages the distinctive fatty acid strengths of each oil: anhydrous butteroil as the primary source of PA, lard for SA, sea buckthorn fruit oil for POA, sunflower oil for LA, and flaxseed oil for ALA, with OA being supplied collectively by multiple sources. Therefore, a feed (namely WB, Western diet with balanced FFAC) for subsequent animal experiments was obtained based on the high-sugar and high-fat Western diet (group W, as reported before [26]) while keeping the proportions of other macronutrients and the total energy density (4.7 kcal/g) unchanged.

### 3.7. FFAC Alleviates Western Diet-Induced Metabolic Dysregulation in Mice

#### 3.7.1. FFAC Substitution Attenuates Western Diet-Induced Weight Gain and Systemic Insulin Resistance

**Figure 7:**
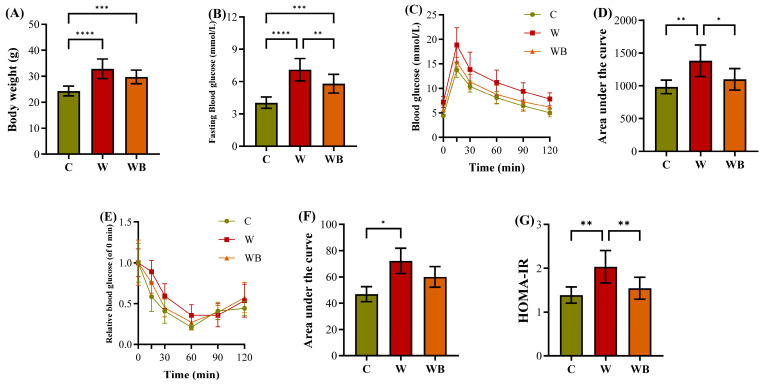
The body weight (**A**), fasting blood glucose (**B**), glucose tolerance curve (**C**) and the area under the curve (**D**), insulin tolerance curve (**E**) and the area under the curve (**F**), as well as Homeostatic Model Assessment for Insulin Resistance (HOMA-IR, (**G**)) of the mice fed by control (group C), Western diet (group W), and Western diet with balanced functional fatty acid component substitution (group WB) (n = 10/group). * *p* < 0.05; ** *p* < 0.01; *** *p* < 0.001; **** *p* < 0.0001.

The 13-week dietary intervention revealed significant effects of diet composition on body weight gain and metabolic parameters in mice (Figure 7A). Consistent with established models of diet-induced obesity, mice fed the high-sugar, high-fat Western diet (W) exhibited the most pronounced body weight, which was significantly higher than that of the control (C) group (32.9 vs. 24.3 g, *p <* 0.0001). The WB diet (Western diet substituted with FFAC) significantly attenuated weight gain (29.8 vs. 32.9 g, *p <* 0.001), indicating that the FFAC replacement effectively mitigated the obesogenic effect arising from the Western diet. Meanwhile, compared with the control group, the weight gain induced by the W was accompanied by profound disruptions in glucose homeostasis, including significant fasting hyperglycemia (7.1 vs. 4.0 mM, *p <* 0.0001, Figure 7B), culminating in a markedly elevated Homeostatic Model Assessment for Insulin Resistance (HOMA-IR) index (2.0 vs. 1.4, *p <* 0.01, Figure 7G). This aligns with extensive literature indicating that high dietary intake of SFA could promote hyperglycemia, hyperinsulinemia, and insulin resistance in key metabolic organs [5,9,16,50]. The W-induced metabolic dysfunction was further confirmed by impaired glucose tolerance, as evidenced by a significantly elevated area under the curve (AUC) during the glucose tolerance test (1383 vs. 984, *p <* 0.01, Figure 7C,D) and the insulin tolerance test (72 vs. 47, *p <* 0.05, Figure 7E,F). Crucially, replacement with the FFAC substantially attenuated these deficits. The WB group showed significantly lower body weight (29.8 vs. 32.9 g), reduced fasting glucose (5.8 vs. 7.1 mM, *p <* 0.01) and a consequent improvement in both HOMA-IR (1.5 vs. 2.0, *p <* 0.01) and the glucose tolerance AUC (1100 vs. 1383, *p <* 0.05), relative to the W group. This recovery suggested that the FFAC replacement enhanced peripheral insulin sensitivity, potentially by enhancing the gluconeogenesis pathway, reducing the lipid-driven inhibition of insulin signaling pathways, and modulating hepatic metabolic pathways [7,18,38].

#### 3.7.2. FFAC Substitution Normalizes Serum Lipid Profile and Prevents Adipose Tissue Expansion

**Figure 8:**
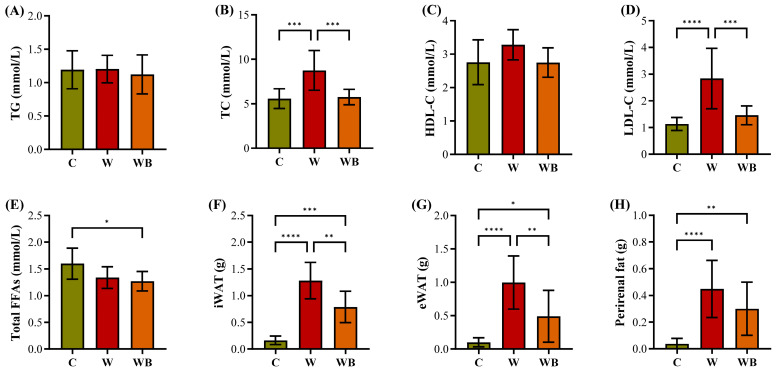
The triglyceride (TG, (**A**)), total cholesterol (TC, (**B**)), high-density lipoprotein-cholesterol (HDL-C, (**C**)), low-density lipoprotein-cholesterol (LDL-C, (**D**)), free fatty acids (FFAs, (**E**)) of serums as well as the weighted white adipose tissue (WAT) of inguinal (iWAT, (**F**)), epididymal (eWAT, (**G**)), and perirenal fat (**H**) of the mice fed by control (group C), Western diet (group W), and Western diet with balanced functional fatty acid component substitution (group WB) (n = 10/group). * *p* < 0.05; ** *p* < 0.01; *** *p* < 0.001; **** *p* < 0.0001.

Consistent with the established link between excessive lipid accumulation and metabolic dysregulation, mice fed the W exhibited significantly elevated serum levels of TC and LDL-C compared to the C group (8.8 mM vs. 5.6 mM and 2.8 mM vs. 1.1 mM, respectively). The critical finding could be summarized as the FFAC replacement completely normalized TC and LDL-C levels (as shown in Figure 8B,D), implying that the reduced lipid absorption thus reduced weight gain in the WB group (Figure 7A), rendering them statistically indistinguishable from those of the C group (5.8 vs. 5.6 mM and 1.5 vs. 1.1, respectively, *p* > 0.05). These data indicated that the specific composition of dietary fat, rather than fat presence alone, is a critical determinant of serum cholesterol homeostasis. Meanwhile, excessive adipose tissue expansion is a key driver of metabolic dysfunction, often triggering chronic inflammation and subsequent insulin resistance in peripheral tissues [26,51]. Our investigation into adipose tissue mass revealed that W led to marked hypertrophy of white adipose tissue (WAT) depots, including inguinal (iWAT), epididymal (eWAT), and perirenal fat (as shown in Figure 8F–H), compared to the C group (1.3 vs. 0.2 g, 1.0 vs. 0.1 g, and 0.4 vs. 0.03 g, respectively, *p <* 0.0001). The WB group exhibited a substantial decrease in the mass of all three WAT depots, compared with the W group. This dissociation between body weight and fat accumulation underscored that the dietary intervention with FFAC specifically prevented the pathological adipose tissue expansion driven by the Western diet. Given that adipose tissue inflammation and dysfunction are strongly correlated with adipocyte hypertrophy, this reduction in fat mass is highly likely to be metabolically beneficial [52]. The significant amelioration of this phenotype in the WB group, coupled with the normalized serum TC and LDL-C levels, collectively indicates a restoration of lipid homeostasis through modified dietary fatty acid composition. Since lipid homeostasis is intrinsically linked to glucose metabolic homeostasis, these improvements suggested the corrected fatty acid ratio not only rectifies dyslipidemia but also lays a potential foundation for enhanced systemic insulin sensitivity [22,23].

#### 3.7.3. FFAC Substitution Mitigates Hepatic Injury and Enhances HDL-Mediated Cholesterol Clearance

**Figure 9:**
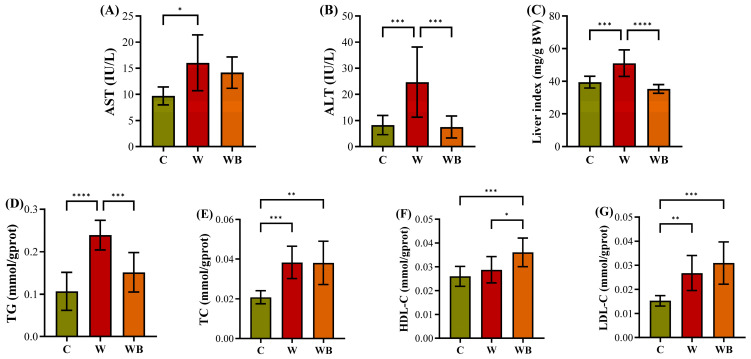
The aspartate aminotransferase (AST, (**A**)) and alanine aminotransferase (ALT, (**B**)) of serums, the weighted liver index (**C**), and the measured triglyceride (TG, (**D**)), total cholesterol (TC, (**E**)), high-density lipoprotein-cholesterol (HDL-C, (**F**)), low-density lipoprotein-cholesterol (LDL-C, (**G**)) in livers of the mice fed by control (group C), Western diet (group W), and Western diet with balanced functional fatty acid component substitution (group WB) (n = 10/group). * *p* < 0.05; ** *p* < 0.01; *** *p* < 0.001; **** *p* < 0.0001.

Consumption of a high-sucrose, high-fat Western diet (W group) resulted in significant hepatic injury, as evidenced by markedly elevated serum levels of the hepatocellular enzymes aspartate aminotransferase (AST, 16.0 vs. 9.7 IU/L, *p <* 0.05) and alanine aminotransferase (ALT, 24.7 vs. 8.2 IU/L, *p <* 0.001) compared with the control (C) group (as demonstrated in Figure 9A,B), indicating hepatocyte membrane damage and necrosis [9,38]. This was further corroborated by a pronounced increase in liver index (51.1 mg/g BW), confirming severe liver function impairment. Biochemically, the W profoundly disrupted lipid homeostasis, leading to hepatic steatosis characterized by elevated TG deposition (0.24 mmol/gprot). In contrast, the WB group, which was fed a diet with FFAC but no other modifications, exhibited a significantly reduced TG content (0.15 mmol/gprot, *p <* 0.0001), indicating that dietary composition beyond fat content critically influenced hepatic lipid accumulation. While TC and LDL-C remained similarly high in both the W and WB groups, a key difference was that the observed HDL-C in the WB group (0.0360 mmol/gprot) was significantly higher than in the C (0.0260 mmol/gprot; *p <* 0.01) and W (0.0287 mmol/gprot; *p <* 0.05) groups. Given HDL-C plays a critical role in reverse cholesterol transport, mediating cholesterol efflux from peripheral tissues to the liver for catabolism and excretion [9,53], this elevation correlated with lower serum TC in the WB group, suggesting that the specific dietary regimen enhances HDL-C function or synthesis. In summary, the Western diet severely perturbs lipid metabolism, promoting steatosis and cellular injury, whereas the WB intervention, while maintaining the same oil ratio, effectively attenuates triglyceride accumulation and enhances the HDL-C-mediated reverse cholesterol transport pathway. Which further alleviated cytotoxicity and endoplasmic reticulum stress and ameliorated systemic inflammation and insulin resistance. This dual effect of reducing lipid deposition and facilitating cholesterol clearance not only mitigates steatosis but also supports the restoration of hepatic insulin sensitivity.

#### 3.7.4. FFAC Substitution Reshapes Gut Microbiota Composition, Notably Enriching *Akkermansia*

**Figure 10:**
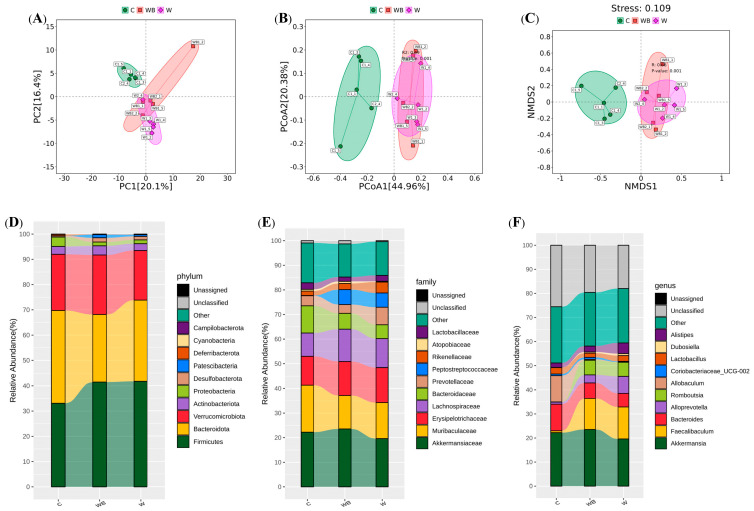
The beta-diversity analyses, including principal component analysis (PCA, (**A**)), principal coordinate analysis (PCoA, (**B**)), and non-metric multidimensional scaling (NMDS, (**C**)) analysis as well as the phylum (**D**), family (**E**), and genus (**F**) levels of intestinal flora in mice fed by control (group C), Western diet (group W), and Western diet with balanced functional fatty acid component substitution (group WB) (n = 5/group).

As a complex community sensitive to dietary inputs, the gut microbiota plays a crucial role in host nutrition and metabolism, including the regulation of glucose and lipid homeostasis [50,54]. Therefore, how different dietary patterns and fatty acid compositions shaped the mouse gut microbiota was further investigated. The beta-diversity analyses (Figure 10) demonstrated clear separation of microbial community structures between the control group and the dietary interventions, with the WB group exhibiting the greatest microbial diversity, encompassing profiles from W and C groups. At the phylum level, high-sugar, high-fat Western diets (i.e., Group W) increased Firmicutes and Bacteroidota, while substitution with FFAC elevated Verrucomicrobiota. Finer taxonomic analysis revealed that, compared to the W group, WB enriched beneficial genera such as *Clostridium_sensu_stricto_1*, *Prevotellaceae_UCG_001*, *Lactobacillus*, and *Alistipes*. A key finding was the significant reduction in the mucin-degrading genus *Akkermansia* (Verrucomicrobiota) in group W, whereas its abundance was restored in group WB. The enrichment of *Akkermansia* in the WB group is particularly noteworthy, as this genus is closely associated with improved glucose metabolism and insulin sensitivity through enhancing intestinal barrier integrity, reducing systemic inflammation, and modulating host signaling pathways that promote insulin action and glucose homeostasis [53,55]. Our findings suggest that dietary fatty acid composition can selectively promote the growth of beneficial *Akkermansia*, offering a microbiota-mediated mechanism for the observed insulin-sensitizing effects [56,57]. Comprehensively, the results indicated that diets high in SFAs adversely affected microbiota composition, while formulations with FFAC replacement promoted a healthier microbial profile, enhancing diversity and enriching taxa associated with improved metabolic outcomes [22,53]. Importantly, the beneficial microbial changes induced by FFAC, such as increased *Akkermansia*, may confer long-term advantages for intestinal homeostasis and metabolic resilience, even in the absence of immediate alterations in lipid accumulation or insulin sensitivity. Thus, the introduced FFAC determinant gut ecosystem structure supports a microbiota configuration linked to better metabolic health.

## 4. Conclusions

This study systematically delineated the distinct and interactive effects of fatty acids on hepatic metabolism, progressing from cellular mechanisms to a whole-animal model. We confirmed that SFAs, notably PA, induce concentration-dependent cytotoxicity. In contrast, UFAs promoted triglyceride storage without compromising viability and showed a reduced propensity to disrupt insulin signaling. Crucially, co-treatment experiments revealed that UFAs could exacerbate PA-induced lipid accumulation while ameliorating its cytotoxic and insulin-desensitizing effects. Synthesizing these findings, we rationally designed a “Functional Fatty Acid Component (FFAC)” with a balanced ratio (PA:SA:POA:OA:LA:ALA = 4:1:1:4:4:1). This FFAC effectively restored insulin sensitivity in HepG2 cells by normalizing the PA-impaired IRS1/PI3K/Akt/GSK3β signaling pathway. Translated into a blended oil formulation and tested in a high-sugar, high-fat diet mouse model, the FFAC substitution significantly mitigated weight gain, systemic insulin resistance, dyslipidemia, and hepatic injury, as well as favorably modulated the gut microbiota, including enriching the beneficial genus *Akkermansia*. Collectively, our findings underscore that the composition of dietary fatty acids, rather than total fat intake alone, is a critical determinant of metabolic health. The rationally designed FFAC presents a promising nutritional strategy for preventing or alleviating diet-induced insulin resistance and its associated metabolic disorders.

## Figures and Tables

**Table 1:** The detected main fatty acid components in six main dietary oils.

Fatty Acid	C14:0	PA C16:0	POA C16:1	SA C18:0	OA C18:1	LA C18:2	ALA C18:3
Anhydrous butteroil	13.7 ± 0.09	40.9 ± 0.02	2.5 ± 0.02	12.9 ± 0.10	26.1 ± 0.09	3.6 ± 0.10	0.4 ± 0.04
Lard	2.0 ± 0.01	30.5 ± 0.67	2.6 ± 0.36	15.7 ± 0.81	37.5 ± 0.00	11.5 ± 0.46	0.2 ± 0.04
Sea buckthorn fruit oil	0	28.0 ± 0.19	23.9 ± 0.13	1.8 ± 0.06	32.5 ± 0.12	12.8 ± 0.22	0.9 ± 0.03
Sunflower oil	0	6.7 ± 0.12	0.1 ± 0.00	3.5 ± 0.05	21.9 ± 0.09	67.6 ± 0.02	0.1 ± 0.00
Flaxseed oil	0	5.7 ± 0.06	0.1 ± 0.00	4.5 ± 0.00	20.1 ± 0.00	15.0 ± 0.01	54.6 ± 0.09
Soybean oil	0.	13.3 ± 2.92	0.1 ± 0.03	3.9 ± 0.26	23.8 ± 1.49	53.0 ± 1.03	5.8 ± 0.16

## Data Availability

The original contributions presented in this study are included in the article. Further inquiries can be directed to the corresponding author.
